# Predicting the Functional, Molecular, and Phenotypic Consequences of Amino Acid Substitutions using Hidden Markov Models

**DOI:** 10.1002/humu.22225

**Published:** 2012-10-03

**Authors:** Hashem A Shihab, Julian Gough, David N Cooper, Peter D Stenson, Gary L A Barker, Keith J Edwards, Ian N M Day, Tom R Gaunt

**Affiliations:** 1 Bristol Centre for Systems Biomedicine and MRC CAiTE Centre, School of Social and Community Medicine, University of BristolBristol, United Kingdom; 2 Department of Computer Science, University of Bristol, The Merchant Venturers BuildingBristol, United Kingdom; 3 Institute of Medical Genetics, School of Medicine, Cardiff UniversityCardiff, United Kingdom; 4 School of Biological Sciences, University of Bristol, Woodland RoadBristol, United Kingdom

**Keywords:** SNP, hidden Markov models, FATHMM

## Abstract

The rate at which nonsynonymous single nucleotide polymorphisms (nsSNPs) are being identified in the human genome is increasing dramatically owing to advances in whole-genome/whole-exome sequencing technologies. Automated methods capable of accurately and reliably distinguishing between pathogenic and functionally neutral nsSNPs are therefore assuming ever-increasing importance. Here, we describe the Functional Analysis Through Hidden Markov Models (FATHMM) software and server: a species-independent method with optional species-specific weightings for the prediction of the functional effects of protein missense variants. Using a model weighted for human mutations, we obtained performance accuracies that outperformed traditional prediction methods (i.e., SIFT, PolyPhen, and PANTHER) on two separate benchmarks. Furthermore, in one benchmark, we achieve performance accuracies that outperform current state-of-the-art prediction methods (i.e., SNPs&GO and MutPred). We demonstrate that FATHMM can be efficiently applied to high-throughput/large-scale human and nonhuman genome sequencing projects with the added benefit of phenotypic outcome associations. To illustrate this, we evaluated nsSNPs in wheat (*Triticum* spp.) to identify some of the important genetic variants responsible for the phenotypic differences introduced by intense selection during domestication. A Web-based implementation of FATHMM, including a high-throughput batch facility and a downloadable standalone package, is available at http://fathmm.biocompute.org.uk.

## Introduction

Nonsynonymous single nucleotide polymorphisms (nsSNPs) lead to amino acid substitutions (AASs) and have the potential to affect the function of the protein product of a gene via the structure, biochemistry and/or splicing of the protein. Advances in high-throughput sequencing technologies have accelerated the rate at which nsSNPs are now being identified [The 1000 Genomes Project, 2010]. Accurate automated computational methods capable of predicting the effects of AASs and amenable to high-throughput analyses of large datasets are therefore of increasing importance for identifying and prioritizing functional nsSNPs for further studies [Thusberg and Vihinen, 2009].

The majority of computational prediction methods utilize evolutionary sequence conservation and/or structural annotations within homologous (orthologous and/or paralogous) proteins from a database of known sequences and/or structures [Ng and Henikoff, 2006]. Traditionally, the BLAST range of pairwise alignment [Altschul et al., 1990] and sequence profile algorithms [Altschul et al., 1997] have been used to search large sequence databases for homologous proteins falling within a predefined similarity threshold. However, weaknesses of these algorithms include the position-invariant scoring matrices in BLAST and the ad hoc estimation of algorithm parameters, that is, position-invariant gap penalties, in PSI-BLAST [Bateman and Haft, 2002]. On the other hand, hidden Markov models (HMMs) [Eddy, 1996; Krogh et al., 1994] are powerful probabilistic models that can be used to capture position-specific information within a multiple sequence alignment (MSA) of homologous sequences. Here, an MSA is represented as a series of match, insert, and delete states linked together via state transitions. A match state models the position-specific amino acid probabilities (with Dirichlet mixtures [Sjölander et al., 1996]) at each column within the sequence alignment whereas insert/delete states allow for particular residues/states to be inserted and skipped, respectively, throughout the sequence alignment (position-specific insertions/deletions). HMM profiles are similar to PSI-BLAST profiles except they are applied within a more rigorous statistical framework and have been shown to perform considerably better when detecting distant relationships between homologous sequences [Madera and Gough, 2002].

Inspired by previous work [Calabrese et al., 2009; Ng and Henikoff, 2001; Thomas et al., 2003], we have capitalized upon recent advances in the HMMER3 software suite [Eddy, 2009] to potentiate the computational prediction of the functional effects of AASs using HMMs. First, we present an unweighted/species-independent method in which homologous sequences are automatically collected and aligned using an iterative search procedure. The resulting MSA is then used to build an ab initio HMM where sequence conservation is then interrogated through the internal match states of the model. In conjunction, sequence conservation within manually curated HMMs representing the alignment of conserved protein domain families: SUPERFAMILY [Gough et al., 2001] and Pfam [Sonnhammer et al., 1997], is interrogated. This additional domain-based analysis is capable of capturing important structural and evolutionary constraints (via priors) that are potentially missed when using an automatically collected alignment of homologous sequences. Next, we introduce a weighted/species-specific method, which incorporates “pathogenicity weights”. These weights are derived from the relative frequencies of disease-associated and functionally neutral AASs mapping onto conserved protein domains. Using a model weighted for human mutations, we obtained performance accuracies that outperformed traditional prediction methods—SIFT, PolyPhen, and PANTHER—on two separate benchmarks. Furthermore, in one benchmark, we achieve performance accuracies that outperform current state-of-the-art prediction methods: SNPs&GO and MutPred. We demonstrate that our method, functional analysis through hidden Markov models (FATHMM), can be efficiently applied to all foreseeable high-throughput large-scale genomic datasets, and advances the field with the added benefit of providing phenotypic outcome associations. In addition to demonstrating the predictive capabilities of FATHMM on multiple benchmarks representing human mutations, we have applied it in practice to a large dataset of nsSNPs in wheat (*Triticum* spp.) to identify some of the key genetic variants responsible for the phenotypic differences introduced by intense selection during domestication and have made this analysis publicly available to the scientific community.

## Materials and Methods

### The Mutation Datasets

A collection of five human mutation datasets from online databases and the literature were downloaded and used in this study (Table 1). First, inherited disease-causing AASs annotated as DMs (damaging mutations) in the Human Gene Mutation Database [Stenson et al., 2009] (HGMD—November 2011; http://www.hgmd.org) and inherited putative functionally neutral AASs in the UniProt database [Apweiler et al., 2004] (UniProt—November 2011; http://www.uniprot.org/docs/humsavar) were downloaded and used to calculate the pathogenicity weights implemented in our weighted/species-specific method. Next, we obtained two human mutation datasets to assess the performance of FATHMM against the performance of other computational prediction algorithms previously reported in the literature: the VariBench database (VariBench—November 2011; http://bioinf.uta.fi/VariBench) used in a comprehensive review [Thusberg et al., 2011] of nine other computational prediction methods [Adzhubei et al., 2010; Bao et al., 2005; Bromberg and Rost, 2007; Calabrese et al., 2009; Capriotti et al., 2006; Li et al., 2009; Mort et al., 2010; Ng and Henikoff, 2001; Ramensky et al., 2002; Thomas et al., 2003] and 267 AASs in four cancer-associated genes (*BRCA1*, *MSH2*, *MLH1*, and *TP53*) used in a recent review [Hicks et al., 2011] of four alternative computational prediction algorithms [Adzhubei et al., 2010; Ng and Henikoff, 2001; Reva et al., 2011; Tavtigian et al., 2006]. Finally, we downloaded a human mutation dataset consisting of disease-associated and putative functionally neutral AASs from the SwissVar portal [Mottaz et al., 2010] (SwissVar—February 2011; http://swissvar.expasy.org) and performed an independent benchmark of FATHMM against eight other computational prediction algorithms [Adzhubei et al., 2010; Calabrese et al., 2009; Capriotti et al., 2006; Ferrer-Costa et al., 2004; Li et al., 2009; Mort et al., 2010; Ng and Henikoff, 2001; Ramensky et al., 2002; Thomas et al., 2003].

**Table 1:** Summary of Mutation Datasets

Dataset	Proteins	Amino acid substitutions	Description
HGMD	2,298	49,532	Inherited disease-causing mutations from HGMD used to calculate our pathogenicity weights
UniProt	11,548	36,928	Inherited putative functionally neutral mutations from UniProt used to calculate our pathogenicity weights
VariBench	9,684	40,470	Benchmarking dataset used in a review of nine alternative computational prediction algorithms [Thusberg et al., 2011]
Hicks et al. 2011	4	267	Benchmarking dataset consisting of both disease-causing and functionally neutral mutations in four well-characterized genes (*BRCA1, MSH2, MLH1, TP53*) used in a recent review of four alternative prediction algorithms [Hicks et al., 2011]
SwissVar	11,986	59,976	Benchmarking dataset used as an independent benchmark of eight alternative prediction algorithms

### Scoring the Magnitude of Effect of Amino Acid Substitutions

The procedure for predicting the functional consequences on the protein function is as follows (see Supp. Fig. S1 for a flow diagram detailing the procedure): the *JackHMMER* component of HMMER3 (one iteration with the optional *–hand* parameter applied; see Supp. Fig. S2) is used to search for homologous sequences within the UniRef90 [Suzek et al., 2007] database (November 2011). As part of this procedure, an ab initio HMM representing the MSA of homologous sequences (with Dirichlet mixtures [Sjölander et al., 1996]) is constructed and used. In conjunction, protein domain annotations from the SUPERFAMILY [Gough et al., 2001] (version 1.75) and Pfam [Sonnhammer et al., 1997] (Pfam-A and Pfam-B; version 26.0) databases are made. The relevant SUPERFAMILY and Pfam HMMs are then extracted only if and when the domain assignment is deemed significant (e-value ≤0.01) and the AAS maps onto a match state within the model.

The information gain (as measured by the Kullback–Leibler [Kullback and Leibler, 1951] divergence from the SwissProt/TrEMBL [Apweiler et al., 2004] amino acid composition) is then calculated at the corresponding match states within the HMMs extracted above. Next, we interrogate the underlying amino acid probabilities modeled by the most informative HMM and assume that a reduction in the amino acid probabilities (when comparing the wild-type to the mutant residue) indicates a potentially negative impact upon protein function whereas a gain in the amino acid probabilities indicates a more favorable substitution. Furthermore, we assume that larger reductions in amino acid probabilities have more substantial effects than smaller reductions in amino acid probabilities. Here, the predicted magnitude of the effect upon protein function is calculated as follows:


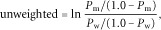
(1)

where *P*_w_ and *P*_m_ represent the underlying probabilities for the wild-type and mutant amino acid residues, respectively.

### Incorporating Species-Specific Pathogenicity Weights

As before, we interrogate the amino acid probabilities within the most informative SUPERFAMILY [Gough et al., 2001] or Pfam [Sonnhammer et al., 1997] (Pfam-A and Pfam-B) HMM (as measured by the Kullback–Leibler [Kullback and Leibler, 1951] divergence from the SwissProt/TrEMBL [Apweiler et al., 2004] amino acid composition). However, for an improved performance in human, the predicted magnitude of effect is weighted by the relative frequency of disease-associated (HGMD) and functionally neutral (UniProt) AASs mapping onto the relevant SUPERFAMILY/Pfam HMM:


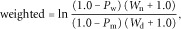
(2)

where *P*_w_ and *P*_m_ represent the underlying probabilities for the wild-type and mutant amino acid residues, respectively, and the pathogenicity weights, *W*_d_ and *W*_n_, represent the relative frequencies of disease-associated and functionally neutral AASs mapping onto the relevant HMM, respectively. The pathogenicity weights also include a pseudo-count of 1.0 to avoid a zero divisible term.

### Annotating the Molecular and Phenotypic Consequences of Amino Acid Substitutions

The overall biological function of a protein is commonly governed by the various combinations of protein domains within it [Peterson et al., 2010]. Therefore, we annotate the potential molecular and phenotypic consequences of pathogenic mutations via domain-centric ontologies [de Lima Morais et al., 2011]. For example, the molecular consequences of AASs are statistically inferred by mapping SUPERFAMILY [Gough et al., 2001] HMMs onto the Gene Ontology [Ashburner et al., 2000]. Moreover, the phenotypic consequences of AASs are annotated by extending these mappings onto several phenotype ontologies including the Human Phenotype Ontology [Robinson et al., 2008], the Mammalian Phenotype Ontology [Smith and Eppig, 2009] and the Plant Phenotype Ontology [Ilic et al., 2007; Pujar et al., 2006].

### Performance Evaluation

In accordance with previous computational prediction methods, the following six parameters (formulae 3-8) were used to assess the performance of our models:


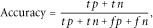
(3)


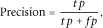
(4)


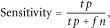
(5)


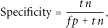
(6)


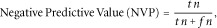
(7)


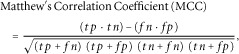
(8)

where *tp* and *fp* refer to the number of true positives and false positives reported and *tn* and *fn* denote the number of true negatives and false negatives reported.

## Results

### Calculating a Prediction Threshold

**Figure 1:**
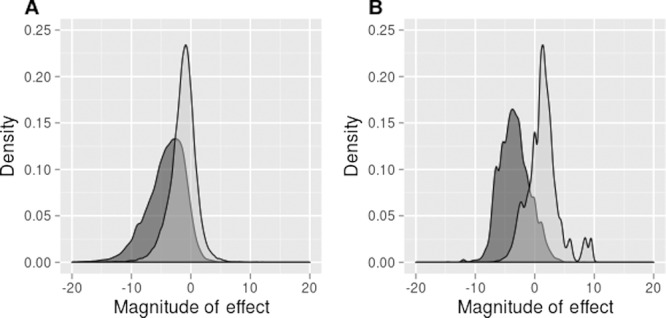
The distribution of the predicted magnitude of effect for disease-associated (shaded region) and functionally neutral (unshaded region) AASs in the SwissVar dataset using our unweighted and weighted methods (**A** and **B**, respectively). From this, we calculated prediction thresholds at which both specificity and sensitivity were maximized (−3.0 and −1.5, respectively).

Theoretically, using our prediction formulae, scores approximately equal to zero indicate that there is no significant change in the underlying amino acid probabilities whereas scores less than zero indicate that an unfavorable substitution has been observed, that is, the mutant residue is less likely to be observed than the wild-type residue, and scores greater than zero indicate that a favorable substitution has been observed, that is, the mutant residue is more likely to be observed than the wild-type residue. However, in practice, FATHMM is sensitive to small fluctuations in the amino acid probabilities modeled within the HMMs. For example, the slightest reduction in amino acid probabilities would yield a pathogenic prediction in our unweighted/species-independent algorithm. Therefore, to eliminate the effects of these fluctuations, we plotted the distribution of the predicted magnitude of effect for both disease-associated and functionally neutral AASs within the SwissVar dataset (Fig. 1). From this, we calculated prediction thresholds for our unweighted and weighted methods at which the specificity and sensitivity were both maximized (−3.0 and −1.5, respectively). Using our unweighted method, we noted that the majority of disease-associated AASs (>60%) fell below our threshold, whereas the majority of functionally neutral polymorphisms (80%) fell above this threshold. Furthermore, using our weighted method, the majority of disease-associated AASs (80%) fell below our threshold whereas a significant proportion of functionally neutral polymorphisms (>80%) fell above this threshold.

### A Performance Comparison Against Published Reviews

The performance of FATHMM was compared against the performance of other computational prediction algorithms reported in two previously published reviews [Hicks et al., 2011; Thusberg et al., 2011]. First, the VariBench database was used to benchmark our method against nine alternative computational prediction algorithms [Adzhubei et al., 2010; Bao et al., 2005; Bromberg and Rost, 2007; Calabrese et al., 2009; Capriotti et al., 2006; Li et al., 2009; Mort et al., 2010; Ng and Henikoff, 2001; Ramensky et al., 2002; Thomas et al., 2003] (Table 2). Typically, the performance of trained/weighted computational prediction algorithms is superior to that of theoretical/unweighted algorithms. Therefore, to allow for a fair comparison to be made, we opted to compare our unweighted/species-independent method against other theoretical/unweighted computational algorithms and our weighted/species-specific method against other trained/weighted computational prediction algorithms. From Table 2, and in terms of performance accuracies, PANTHER [Thomas et al., 2003] appears to be the best performing theoretical/unweighted prediction method with an accuracy of 76%. It appears that both SIFT [Ng and Henikoff, 2001] (another sequence-based method) and our unweighted method perform less favorably with accuracies of 65% and 69%, respectively, indicating that FATHMM is somewhat the better option of the two. The observed performances in our analysis indicate that our weighted method is the best performing method available with an overall performance accuracy of 86%, thereby outperforming the current state-of-the-art prediction methods MutPred [Li et al., 2009; Mort et al., 2010] (81%) and SNPs&GO [Capriotti et al., 2006] (82%).

**Table 2:** Performance of Computational Prediction Methods using the VariBench Benchmarking Dataset

	*tp*	*fp*	*tn*	*fn*	Accuracy^a^	Precision^a^	Specificity^a^	Sensitivity^a^	NVP^a^	MCC^a^
Theoretical/unweighted computational prediction methods										
SIFT	10,464	4,856	12,188	7,433	0.65	0.64	0.62	0.68	0.66	0.30
PolyPhen 1^b^	10,093	9,185	17,669	3,199	0.69	**0.77**	**0.85**	0.52	0.64	0.39
PolyPhen 1^c^	14,285	4,993	13,671	7,197	0.70	0.68	0.66	0.74	0.72	0.40
PANTHER	9,689	2,859	8,676	2,797	**0.76**	0.76	0.76	**0.77**	**0.77**	**0.53**
FATHMM (unweighted)	11,561	4,839	16,257	7,707	0.69	0.72	0.77	0.60	0.66	0.38
Trained/weighted computational prediction methods										
PolyPhen 2^b^	13,807	5,102	13,863	6,010	0.71	0.71	0.70	0.73	0.72	0.43
PolyPhen 2^c^	16,206	2,703	10,199	9,674	0.69	0.64	0.51	0.86	0.78	0.39
PhD-SNP	11,900	6,896	16,788	4,377	0.71	0.75	0.79	0.63	0.68	0.43
SNPs&GO	13,736	5,487	17,028	1,382	0.82	**0.90**	**0.92**	0.71	0.76	0.65
nsSNPAnalyzer	4,360	2,778	1,319	943	0.60	0.59	0.58	0.61	0.60	0.19
SNAP	16,000	2,146	8,190	6,387	0.72	0.67	0.56	**0.88**	0.83	0.47
MutPred	13,829	2,507	15,891	4,557	0.81	0.79	0.78	0.85	0.84	0.63
FATHMM (weighted)	14,231	1,633	10,146	2,336	**0.86**	0.86	0.86	0.86	**0.86**	**0.72**

*tp*, *fp*, *tn*, *fn* refer to the number of true positives, false positives, true negatives, and false negatives, respectively.

a Accuracy, Precision, Specificity, Sensitivity, NVP, and MCC are calculated from normalized numbers.

b “Probably Pathogenic” predictions classed as disease causing.

c “Probably Pathogenic” predictions classed as functionally neutral.

The performances of alternative computational prediction algorithms have been reproduced with permission from Thusberg et al. (2011). Copyright 2012, Wiley.

Next, we used the Hicks dataset to benchmark FATHMM against four other computational prediction algorithms (using their native alignments) [Adzhubei et al., 2010; Ng and Henikoff, 2001; Reva et al., 2011; Tavtigian et al., 2006] (Table 3). Overall, Align-GVGD [Tavtigian et al., 2006] appears to be the best performing method. However, Align-GVGD employs gene-specific alignments and its performance is severely affected when automatically generated alignments are used [Hicks et al., 2011]. These results appear to indicate that our unweighted method is more specific than either Align-GVGD or SIFT; however, we also noted higher false positive rates when compared with the other prediction methods. In general, and perhaps more surprisingly, it appears that the performance of all trained/weighted computational prediction methods is inferior across the four genes when compared to their theoretical/unweighted counterparts. Again, although no one trained/weighted prediction method performs best across the four genes, it would appear that our weighted method is, on average, the most specific/least sensitive.

**Table 3:** Specificity and Sensitivity of Computational Prediction Methods in Four Well-Characterized Genes (*BRCA1*, *MSH2*, *MLH1*, and *TP53*)

	*BRCA1*		*MSH2*		*MLH1*		*TP53*	
				
Algorithm	Specificity	Sensitivity	Specificity	Sensitivity	Specificity	Sensitivity	Specificity	Sensitivity
Theoretical/unweighted computational prediction methods								
SIFT	0.31	**0.94**	0.46	**0.90**	0.52	0.72	0.75	**0.84**
Align-GVGD	**0.94**	0.71	0.55	**0.90**	0.52	**0.97**	**1.00**	0.82
FATHMM (unweighted)	0.56	0.65	**0.73**	0.84	**0.71**	0.77	**1.00**	0.71
Trained/weighted computational prediction methods								
PolyPhen-2	0.38	0.77	0.36	0.90	**0.67**	0.90	**1.00**	0.84
X-Var	0.56	**0.82**	0.27	**1.00**	0.33	**1.00**	0.50	0.96
FATHMM (weighted)	**0.70**	0.47	**0.50**	0.79	0.24	0.97	NA^a^	**1.00**

a The specificity for our weighted method in this instance is uninformative as there was only one neutral mutation falling within conserved protein domains.

The performances of alternative computational prediction algorithms have been reproduced with permission from Hicks et al. (2011). Copyright 2012, Wiley.

**Table 4:** Performance of Computational Prediction Methods Using the SwissVar Benchmarking Dataset

	*tp*	*fp*	*tn*	*fn*	Accuracy^a^	Precision^a^	Specificity^a^	Sensitivity^a^	NVP^a^	MCC^a^
Unweighted computational prediction methods										
SIFT	15,634	6,318	28,236	7,716	**0.74**	**0.79**	**0.82**	0.67	0.71	**0.49**
PolyPhen 1	12,803	8,759	18,603	4,497	0.71	0.70	0.68	**0.74**	**0.72**	0.42
PANTHER	8,283	5,842	17,447	5,162	0.68	0.71	0.75	0.62	0.66	0.37
FATHMM (unweighted)	14,311	6,717	29,454	9,429	0.71	0.76	0.81	0.60	0.67	0.43
Weighted/trained computational prediction methods										
PolyPhen 2 (HumDiv)	19,782	13,592	20,874	3,204	0.73	0.69	0.61	0.86	0.81	0.48
PolyPhen 2 (HumVar)	19,928	13,239	21,227	3,058	0.74	0.69	0.62	0.87	0.82	0.50
PhD-SNP Sequence	15,695	9,380	26,838	8,062	0.70	0.72	0.74	0.66	0.69	0.40
PhD-SNP Profile	17,548	7,233	27,731	5,161	0.78	0.79	0.79	0.77	0.78	0.57
PMut	13,498	12,156	23,636	10,159	0.62	0.63	0.66	0.57	0.61	0.23
SNPs&GO	17,768	3,768	29,101	5,655	0.82	0.87	0.89	0.76	0.79	0.65
MutPred	21,365	3,500	32,719	2,392	**0.90**	**0.90**	**0.90**	**0.90**	**0.90**	**0.80**
FATHMM (weighted)	15,916	3,017	19,713	4,496	0.82	0.85	0.87	0.78	0.80	0.65

*tp*, *fp*, *tn*, *fn* refer to the number of true positives, false positives, true negatives, and false negatives, respectively.

a Accuracy, Precision, Specificity, Sensitivity, NVP, and MCC are calculated from normalized numbers.

### An Independent Benchmark Against Other Computational Prediction Methods

**Figure 2:**
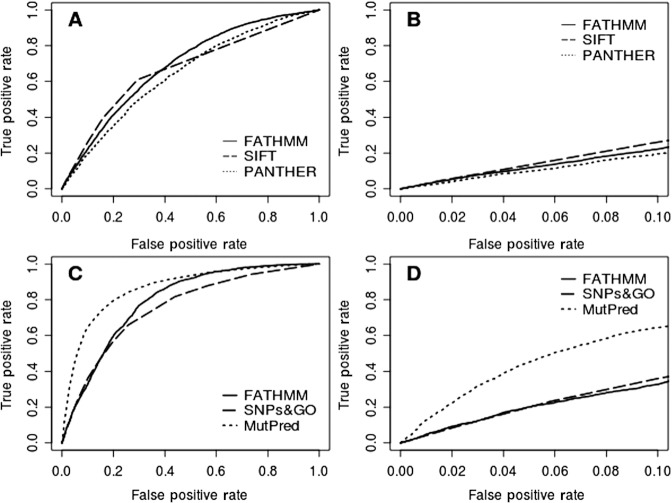
Receiver operating characteristic (ROC) curves for the top-ranking computational prediction algorithms evaluated using the SwissVar dataset. Here, we compare our unweighted method against SIFT and PANTHER (**A**—full curve; **B**—10% false positive rate) whereas our weighted method is compared to SNPs&GO and MutPred (**C**—full curve; **D**—10% false positive rate). Full ROC curves for all computational prediction algorithms evaluated are made available in Supp. Figure S3.

Although we recognize the importance of comparing prediction methods in relation to previously established benchmarks, we also conducted our own benchmark (using the SwissVar mutation dataset – see Materials & Methods) comparing the performance of FATHMM against eight published computational prediction methods [Adzhubei et al., 2010; Calabrese et al., 2009; Capriotti et al., 2006; Ferrer-Costa et al., 2004; Li et al., 2009; Mort et al., 2010; Ng and Henikoff, 2001; Ramensky et al., 2002; Thomas et al., 2003] (Table 3—see Supp. Table S1). In contrast to the VariBench benchmark, and in terms of performance accuracies, it appears that both SIFT [Ng and Henikoff, 2001] and our own unweighted method outperform PANTHER [Thomas et al., 2003] (68%) with performance accuracies of 74% and 71%, respectively, indicating that SIFT is somewhat the better option. The best performing method is MutPred [Li et al., 2009; Mort et al., 2010] with a performance accuracy of 90%. However, the observed performances show that our weighted method once again performs favorably when compared to other state-of-the-art prediction methods: SNPs&GO [Calabrese et al., 2009], despite the domain-based restriction inherited from our pathogenicity weights. Next, we compared the performance of our unweighted method via receiver operating characteristic (ROC) curves against the top ranking theoretical/unweighted computational prediction methods: SIFT and PANTHER (Fig. 2A, B—see Supp. Fig. S3 for a comprehensive ROC curve against all evaluated methods). Impressively, given a 10% false positive rate, it seems that the performance of our unweighted method is comparable to SIFT thereby highlighting the sensitivity of our method to small fluctuations within the underlying amino acid probabilities. Furthermore, we compared the performance of our weighted method via ROC curves against the top-ranking trained/weighted prediction algorithms: MutPred and SNPs&GO (Fig. 2C, D). These results confirm that our weighted method performs favorably when compared to SNPs&GO.

The pathogenicity weights incorporated in FATHMM were not directly used to train for, or recognize, pathogenic sequences and/or mutations. We do nevertheless recognize the potential for bias in the performances observed. Therefore, to remove this bias, we performed a “leave-one-out” analysis on all benchmarking datasets. Here, we adjusted our pathogenicity weights, *W*_d_ and *W*_n_, if and only when the AAS being evaluated was present in either the HGMD [Stenson et al., 2009] or UniProt [Apweiler et al., 2004] datasets. We observed no significant deviations in the performance measures reported above and hence concluded that the performances observed were not biased toward the pathogenicity weights employed (see Supp. Table S2).

**Figure 3:**
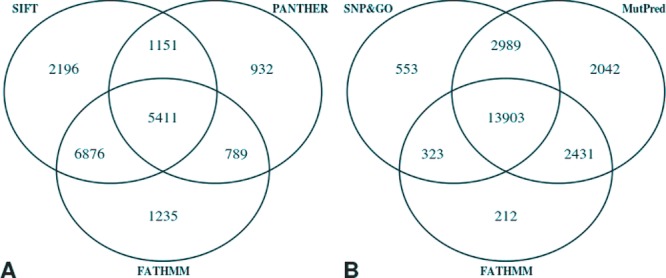
The intersection of disease-associated amino acid substitutions correctly identified by the top-ranking computational prediction algorithms evaluated using the SwissVar dataset. Here, we compare our unweighted method against SIFT and PANTHER (**A**) whereas our weighted method is compared to SNPs&GO and MutPred (**B**).

To understand the potential complementarity/redundancy of FATHMM to other methods, we assessed the intersection of disease-associated AASs correctly identified (true positives) by our method and the top-ranking computational prediction algorithms (Fig. 3). From this analysis, it was clear that no one method completely encapsulates all other methods that is, each method succeeded in correctly and uniquely identifying some disease-associated AASs where other methods did not. These results reaffirm previous suggestions that combining predictions from multiple prediction methods has the potential to perform better than any individual method [Liu et al., 2011; Olatubosun et al., 2012].

### Facilitating the High-Throughput Analysis of Large Genomic Datasets

Anticipating a massive increase in the number of available whole-genome and whole-exome datasets, the need for accurate computational prediction methods capable of processing these datasets in a timely fashion is increasingly apparent. As a result, the majority of computational prediction algorithms now offer some form of precomputed facility allowing for near-instant predictions to be returned (see Supp. Table S3). However, only SIFT [Ng and Henikoff, 2001] and PolyPhen-2 [Adzhubei et al., 2010] allow for batch submissions (with restrictions) to be made. To facilitate the high-throughput analysis of large-scale genomic datasets, our public Web-server provides up-to-date (precomputed) domain assignments for several large sequence collections, including SwissProt/TrEMBL [Apweiler et al., 2004]; thereby enabling (unrestricted) near-instant predictions to be made for AASs falling within conserved protein domains. Furthermore, our precomputed database is available as an optional download enabling near-instant predictions to be made while running our software locally.

### Annotating Phenotypic Outcome Associations

As previously alluded to, FATHMM not only predicts the potentially deleterious nature of AASs but is also capable of annotating the molecular and phenotypic consequences of these mutations via domain-centric ontologies. To illustrate this, we evaluated the predicted phenotypic consequences of disease-associated AASs within the SwissVar dataset (Supp. Table S4). As expected, the phenotypic consequences of well-characterized diseases are correctly identified. For example, the cardiovascular consequences of the C1971Y mutation in *FBN1* (Marfan syndrome; MIM# 154700) are correctly identified via domain-based ontological associations. However, potential issues of using domain-centric ontologies arise when a common domain harbors multiple mutations with distinct and uniquely expressed phenotypes. In these instances, domain-centric ontological associations may have become diluted and should therefore be used with caution. For example, the predicted phenotypic consequences for the R239C mutation in *CHRNG* (Escobar syndrome; MIM# 265000) are consistent with the associated syndrome, which is characterized by a decrease in fetal movement and overall muscle weakness. However, phenotypes not associated with (or secondary to) Escobar syndrome, for example abnormalities in temperature regulation, were also predicted. Nevertheless, we foresee that these annotations will be most prominent in protein sequences of unknown function and/or ongoing nonhuman genome sequencing projects, as demonstrated below.

### Case Study: Annotating the Functional and Phenotypic Consequences of nsSNPs in Wheat

As the world's population continues to grow, so does the demand for crops with particular characteristics such as drought resistance, high yield and resistance to pests and pathogens. The cultivation and repeat harvesting of wild “landrace” wheat varieties has led over time to the emergence of domesticated “elite” wheat varieties with desirable phenotypic characteristics. In an attempt to elucidate some of the important genetic variants responsible for these characteristics, we collected single nucleotide variants (SNVs) from four elite UK bread wheat varieties (Avalon, Cadenza, Rialto, and Savannah) and have predicted the functional effects of these mutations when compared to four landrace wheat varieties from the Watkins collection held at the John Innes Centre, Norwich, UK (304, 306, 311, and 328). For this analysis, SNVs were mapped onto the draft wheat genome assembly and six-frame translated. For each reading frame, SUPERFAMILY [Gough et al., 2001] and Pfam [Sonnhammer et al., 1997] domain assignments on the full-length amino acid sequence were made and the corresponding AASs were evaluated using our unweighted method. We found several biologically interesting SNV differences between the landrace and elite wheat varieties (see Supp. Table S5). For example, wheat contig F0Z7V0F01D2DA5 had a SNP at position 172 in the casein kinase II beta subunit domain with phenotypic consequences predicted to affect the flower developmental stages and vegetative growth. The casein kinase II beta subunit domain has a putative function in flowering time regulation in the model plant *Arabidopsis* [Ogiso et al., 2010] and is likely to be biologically significant as European domestic wheat will have been selected to grow under shortened seasons and different day lengths to the landraces. Next, wheat contig GIZP4PP04H5FGF had a SNP at position 219, which lies within the Pfam starch synthase catalytic domain. Once again, this is likely to be biologically significant as the quantity and properties of starch are important to the baking properties of cultivated wheat and will thus have been under strong selection. Finally, wheat contig 09781 had a SNP at position 368 in the cysteine proteinase domain with predicted phenotypic consequences affecting plant structure development. In cereals, cysteine proteases are known to be important in the laying down of storage proteins [Fahmy et al., 2004]. As with starch, the properties of wheat storage proteins will have come under intense selection during domestication as they are the most important determinant of baking qualities and economic yield. These results, made publicly available to the wheat genomics community at http://www.cerealsdb.uk.net/functional_snps/index.htm, illustrate the potential additional utility of FATHMM in predicting the functional consequences of variants identified in ongoing nonhuman genome sequencing projects (even in species very distantly related to human).

## Discussion

Here, we have introduced and discussed the FATHMM software and server: a species-independent method with optional species-specific weightings for the prediction of the functional effects of protein missense variants. Inspired by previous sequence-based computational prediction algorithms [Ng and Henikoff, 2001; Thomas et al., 2003], our unweighted/species-independent method interrogates sequence conservation through the underlying amino acid probabilities modeled by the internal match states of several HMMs representing the alignment of homologous sequences and conserved protein domains. Following a similar weighting scheme implemented in SNPs&GO [Calabrese et al., 2009], our weighted/species-specific method amalgamates sequence conservation within the HMMs with “pathogenicity weights” representing the relative frequencies of disease-associated and functionally neutral AASs mapping onto conserved protein domains. The pathogenicity weights incorporated here are not directly used to train for, or recognize, pathogenic sequences, and/or mutations. Instead, these weights are capable of recognizing protein domains (species-independent/evolutionary units) sensitive to or intolerant of missense mutations. Therefore, the pathogenicity weights implemented in FATHMM are also likely to represent an improvement for nonhuman organisms (especially those not too distantly related to human) [Ferrer-Costa et al., 2005].

The performance of FATHMM was compared to the performances of alternative computational prediction methods previously reported in two published reviews [Hicks et al., 2011; Thusberg et al., 2011]. Furthermore, we performed our own independent benchmark comparing the performance of FATHMM against the performance of other computational prediction methods. In two benchmarks (VariBench/SwissVar), the performance of our unweighted method is comparable to another sequence-based method: SIFT [Ng and Henikoff, 2001], and to a sequence/structure-based method: PolyPhen-1 [Ramensky et al., 2002]. This performance reaffirms the ability of FATHMM to recognize important structural and/or evolutionary constraints (via priors) modeled within manually curated HMMs representing the alignment of conserved protein domains: SUPERFAMILY [Gough et al., 2001] and Pfam [Sonnhammer et al., 1997]. A detailed analysis of four cancer-associated genes (Hicks; *BRCA1*, *MSH2*, *MLH1*, and *TP53*) shows Align-GVGD [Tavtigian et al., 2006] to be the best performing prediction method. However, this can be attributed to the manually curated (gene-specific) sequence alignments employed in the prediction method. On average, the performance of our unweighted method in this benchmark is comparable to SIFT.

An important issue to consider when comparing the performance of trained/weighted computational prediction methods is the cross-validation dataset, that is, these prediction methods should ideally be tested using “blind” datasets to minimize the bias in the performances observed. Unfortunately, this level of testing is not possible as it would require retraining/validating all prediction methods with common datasets. However, the majority of disease-associated AASs in the VariBench database were collected from Locus-Specific Databases (LSDB) and are not found in commonly used training datasets, for example, SwissProt/TrEMBL [Apweiler et al., 2004]. Therefore, the curators claim this bias is minimized in this dataset [Thusberg et al., 2011]. Here, the performance of our weighted method appears to outperform the current state-of-the-art prediction methods: MutPred [Li et al., 2009; Mort et al., 2010] and SNPs&GO [Calabrese et al., 2009]. By contrast, the mutation dataset used in our independent benchmark was collected from the SwissVar [Mottaz et al., 2010] portal. As a result, the estimated performances of other computational prediction methods which have been trained on SwissProt/TrEMBL mutations may be overinflated. Here, MutPred is the best performing method; however, the performance of our weighted method is comparable to SNPs&GO. To alleviate the potential bias in our method, we performed a leave-one-out analysis and found no significant deviations in the observed performances; we therefore concluded that the performances observed in FATHMM were not an artifact of the weighting scheme employed. The performances of all trained/weighted computational prediction algorithms were, somewhat surprisingly, inferior when compared to their theoretical/unweighted counterparts across four cancer-associated genes. The performances observed within Align-GVGD (gene-specific alignments) suggest that there may be some benefit in incorporating disease-specific weightings into our algorithm, for example, cancer-specific weightings similar to those employed by Capriotti and Altman (2011).

A potential disadvantage of our weighted method is the inherited restriction (via the weighting scheme employed) to AASs falling within conserved protein domains. However, protein domain annotations from the SUPERFAMILY and Pfam databases encompass around 80% of the SwissProt/TrEMBL database [Punta et al., 2012]. In our analysis, we were able to analyse a large proportion (>70%) of the VariBench and SwissVar benchmarking datasets. On the other hand, unlike other sequence-based prediction methods (including our own unweighted method), which are too slow for practical use in large-scale sequencing projects, our weighted method uses computationally inexpensive domain assignments. Therefore, FATHMM can be efficiently applied to all foreseeable high-throughput large-scale genomic datasets with minimal reduction in coverage. In addition, our method advances the field with its unique ability to annotate the molecular and phenotypic consequences of AASs using several domain-centric ontologies [de Lima Morais et al., 2011] including the Human Phenotype Ontology [Robinson et al., 2008] and the Mammalian Phenotype Ontology [Smith and Eppig, 2009]. Thus, by coupling the functional predictions generated by FATHMM with domain-based ontological associations, as opposed to protein level annotations, we have developed a tool, which is capable of providing useful insights into the underlying mechanisms disrupted by AASs without any prior/background information on the protein itself. A Web-based implementation of FATHMM, which facilitates the high-throughput analysis of large-scale genomic datasets, and includes a downloadable open-source software package, is available at http://fathmm.biocompute.org.uk.

## Supplementary material

Disclaimer: Supplementary materials have been peer-reviewed but not copyedited.
