# Supplementary material for: Predicting the Functional, Molecular, and Phenotypic Consequences of Amino Acid Substitutions using Hidden Markov Models
Source: Hum Mutat. 2012 Oct 3;34(1):57–65. doi: 10.1002/humu.22225 (PMC3558800; doi:10.1002/humu.22225)
Supplement: Supplementary file 1 [file humu0034-0057-SD1.pdf]

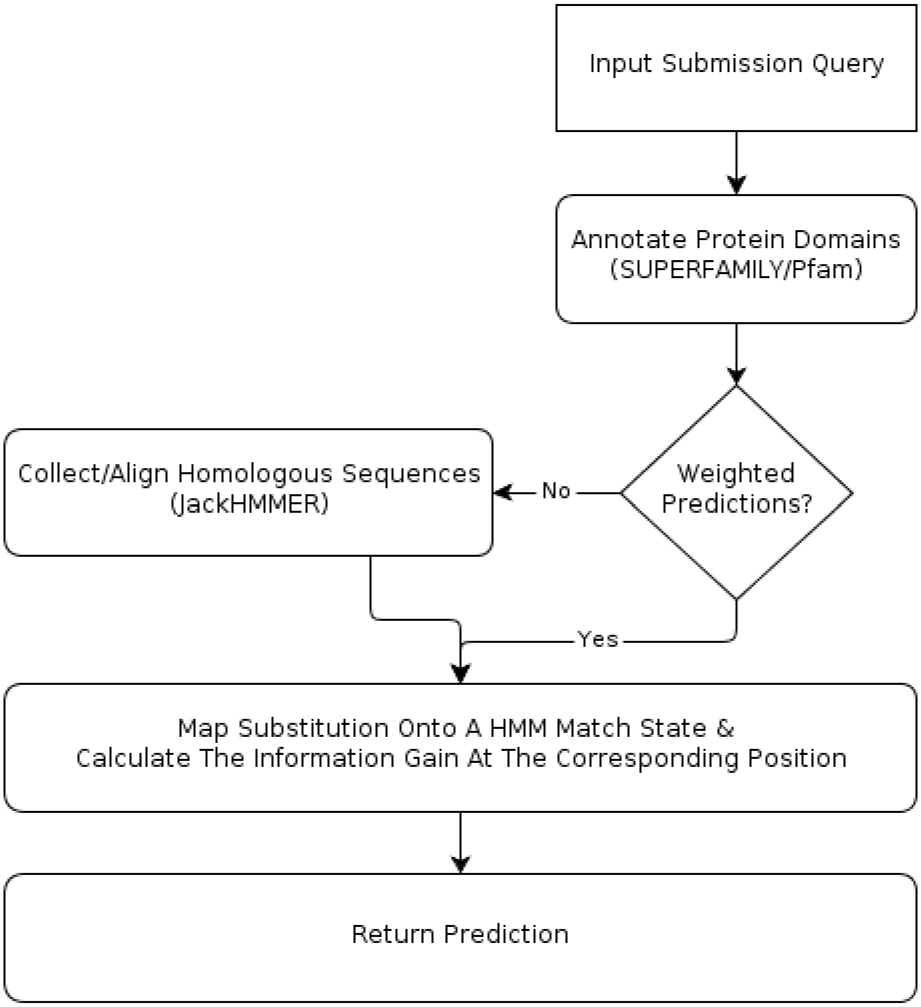

Supp. Figure S1. Scoring the Magnitude of Effect of Amino Acid Substitutions. The expected input for an unweighted prediction is the protein sequence and substitution whereas the expected input for a weighted prediction is the SwissProt/TrEMBL protein ID and substitution. Next, protein domain annotations from the SUPERFAMILY and Pfam databases are made. In addition, if an unweighted prediction is requested, an *ab initio* HMM is built from the alignment of homologous sequences collected as part of the *JackHMMER* algorithm. The amino acid substitution is then mapped onto the corresponding HMM match states where the information gain (as measured by the Kullback-Leibler divergence from the SwissProt/TrEMBL amino acid composition) is then calculated. This is then used to deduce the most informative HMM and a prediction is made accordingly.

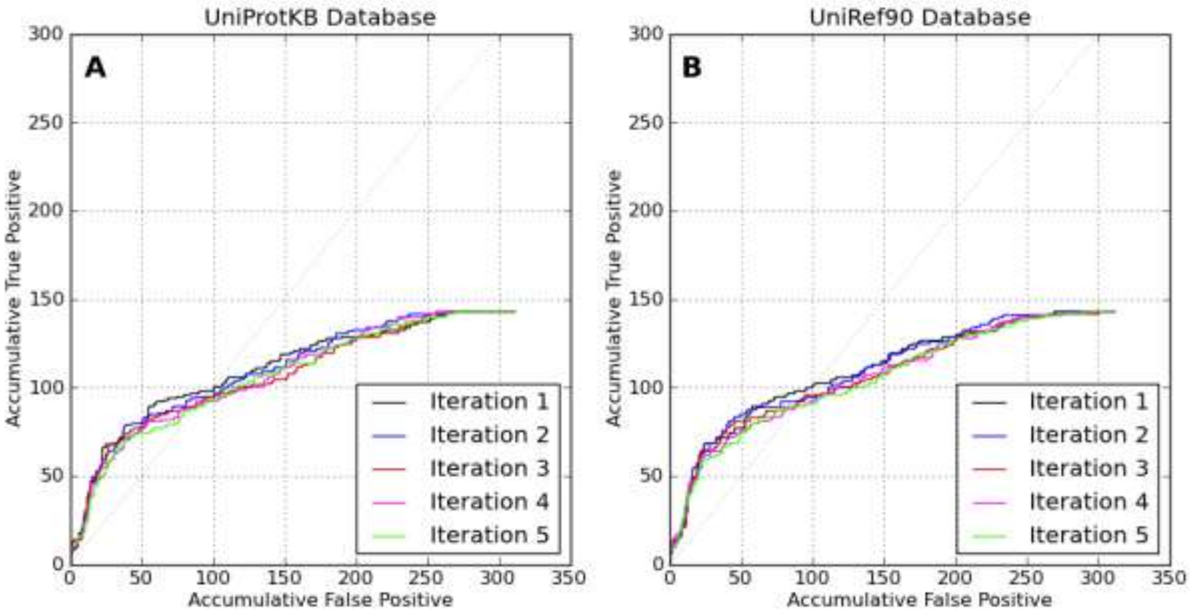

Supp. Figure S2. In this study, we interrogate the amino acid conservation within homologous (both orthologous and paralogous) sequences using HMMs. The *JackHMMER* algorithm takes a query sequence and iteratively searches a sequence database for homologous sequences (akin to the PSI-BLAST algorithm). In order to establish the optimal parameters for the *JackHMMER* algorithm, i.e. the sequence database to search (SwissProt/TrEMBL or UniRef90) and the number of iterations required, we randomly sampled 100 proteins from the SwissVar dataset (involving 143 disease-causing and 311 functionally neutral AASs) and compared the performance of FATHMM at each *JackHMMER* iteration using Equation 1 (Figures A & B). In general, we observed no significant improvements in the performance of FATHMM at later iterations in either sequence database, indicating that a single iteration is sufficient when searching for homologous sequences for the computational prediction of the functional effects of amino acid substitutions. Therefore, the final version of FATHMM implements one *JackHMMER* iteration across the UniRef90.

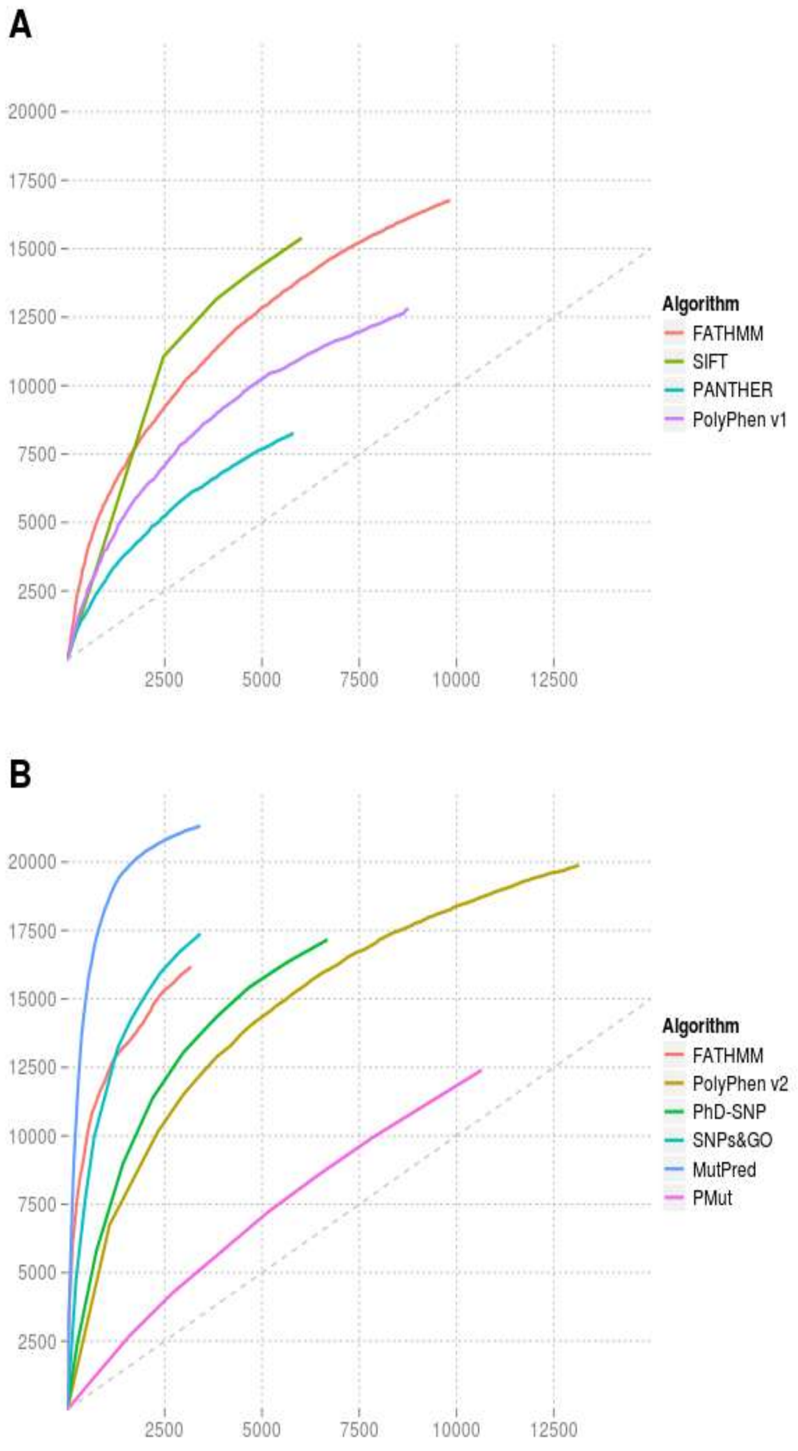

Supp. Figure S3. A Receiver Operating Characteristic (ROC) curve showing the accumulated true positives plotted against the accumulated false positives for all unweighted (A) and weighted (B) computational prediction algorithms evaluated using the SwissVar benchmark dataset. Here, the "HumVar" and "Profile" versions of PolyPhen v2 and PhD-SNP are plotted as they performed best (in terms of performance accuracy).

Supp. Table S1. Mutation Submission Procedure for Computational Prediction Methods

Prediction Method	Methodology
SIFT	Local Installation
PolyPhen 1	Automatic Web Submission/Scraping
PolyPhen 2	Batch Submission
PANTHER	Local Installation
PhD-SNP	Local Installation
PMut	Author Request
SNPs&GO	Automatic Web Submission/Scraping
MutPred	Author Request

For methods without a batch submission/download facility, we developed custom web-scraping scripts in the Python programming language (available upon request) which submitted the mutations, one at a time, and parsed the predictions. For prediction methods where this was not possible, e.g. MutPred, the authors of the method kindly processed our mutation dataset.

Supp. Table S2. Performance of Our Weighted Method using a Leave-One-Out Analysis

	Accuracy	Precision	Specificity	Sensitivity	NVP	MCC
VariBench†	0.86	0.86	0.86	0.85	0.85	0.71
SwissVar†	0.81	0.84	0.85	0.77	0.79	0.63
BRCA1	-	-	0.60	0.47	-	-
MSH2	-	-	0.50	0.74	-	-
MLH1	-	-	0.19	0.95	-	-
TP53	-	-	NA	1.00	-	-

For this analysis, we adjusted our pathogenicity weights, W_d and W_n, if and only when the AAS being evaluated was present in either the HGMD [Stenson et al., 2009] or UniProt [Apweiler et al., 2004] datasets. We observed no significant deviations in the performance measures and concluded that the performances observed in our benchmarks were not biased towards the pathogenicity weights employed.

† The performance measurements reported are calculated from normalised numbers.

Supp. Table S3. Availability of Computational Prediction Methods

	SIFT	PolyPhen v1	PolyPhen v2	PANTHER	PhD-SNP (Profile)	PMut	SNPs&GO	MutPred	FATHMM
Web-Server	✓	*	✓	✓	✓	✓	✓	✓	✓
Average Run-Time (Single Query)	†	-	†	< 1 Minute	2 Minutes	†	†	†	†
Batch Facility Available	✓	-	✓	✗	✗	✗	✗	✗	✓
Batch Facility Limitation	1,000 Proteins	-	150,000 AASs	-	-	-	-	-	Unlimited
Phenotypic Associations	✗	-	✗	✗	✗	✗	✗	✗	✓
Download Available	✓	-	✓	✓	✓	✗	✗	✗	✓
Optional Pre-Computed Database‡	✗	-	✓	✗	✗	-	-	-	✓
Open Source	✗	-	✗	✓	✗	-	-	-	✓

* PolyPhen v1 has now been discontinued and is no longer accepting user submissions
† pre-computed / near-instant predictions available (restrictions may apply)
‡ optional pre-computed database for near-instant predictions while running locally

Supp. Table S4. The Predicted Phenotypic Consequences of Disease-Associated AASs against their Associated Diseases/Abnormalities

Gene &<br>Amino Acid Substitution	Associated Disease &<br>MIM Identifier	Phenotypic Inference
FBN1<br>C1971Y	Marfan syndrome<br>MIM# 154700	⅄ Dilatation of the Ascending Aorta<br>⅄ Abnormality of the Aortic Valve<br>⅄ Emphysema<br>⅄ Mitral Regurgitation
HEXA<br>W485R	GM2-Gangliosidosis type 1<br>MIM# 272800	⅄ Abnormality of Metabolism/Homeostasis<br>⅄ Angiokeratoma<br>⅄ Mental Deterioration<br>⅄ Cardiomegaly<br>⅄ Beaking of Vertebral Bodies
PSAP<br>C388F	Atypical Gaucher disease<br>MIM# 610539	⅄ Abnormality of the Musculoskeletal System<br>⅄ Abnormality of Metabolism/Homeostasis<br>⅄ Abnormality of the Immune System<br>⅄ Functional Respiratory Abnormality<br>⅄ Abnormality of the Lung<br>⅄ Respiratory Insufficiency
CHRNG<br>R239C	Escobar syndrome<br>MIM# 265000	⅄ Intermittent Episodes of Respiratory Insufficiency Due to Muscle Weakness<br>⅄ Prolonged Miniature Endplate Currents<br>⅄ Decreased Size of Nerve Terminals<br>⅄ Generalized Muscle Weakness due to Defect at the Neuromuscular Junction<br>⅄ Muscle Fiber Atrophy<br>⅄ Multiple Pterygia<br>⅄ Generalized Amyoplasia<br>⅄ Decreased Fetal Movement<br>⅄ Hypoplastic Heart<br>⅄ Cystic Hygroma<br>⅄ Poor Feeding due to Muscle Weakness<br>⅄ Easy Fatigability<br>⅄ Gower Sign<br>⅄ Flat Nose<br>⅄ Abnormal Cervical Curvature<br>⅄ Thin Ribs<br>⅄ Vertebral Fusion<br>⅄ Weak Cry<br>⅄ Ophthalmoparesis<br>⅄ Ptosis<br>⅄ High-Arched Palate<br>⅄ Poor Suck<br>⅄ Macrotia<br>⅄ Bulbar Palsy<br>⅄ Intrauterine Growth Restriction<br>⅄ Myopathy<br>⅄ Malignant Hyperthermia<br>⅄ Umbilical Hernia<br>⅄ Joint Dislocation<br>⅄ Abnormality of Temperature Regulation<br>⅄ Abnormality of Prenatal Development or Birth<br>⅄ Abnormality of Muscle Fibers<br>⅄ Abnormality of the Nervous System<br>⅄ Functional Respiratory Abnormality

Supp. Table S5. Interesting Single Nucleotide Variants (SNVs) Between the "Elite" and "Landrace" Wheat Varieties

Wheat Contig & nsSNP Position	Phenotypic Inference
Contig F0Z7V0F01D2DA5<br>nsSNP Position 127	⅄ 1 Main Shoot Growth<br>⅄ Seed Development Stages<br>⅄ Plant Structure Development Stage<br>⅄ Flower Development Stages<br>⅄ Corolla Developmental Stages<br>⅄ 4 Anthesis<br>⅄ 3 Flower Organ Development Stages<br>⅄ 4 Leaf Senescence Stage<br>⅄ A Vegetative Growth<br>⅄ Embryo Development Stages<br>⅄ Whole Plant Growth Stage<br>⅄ Leaf Production<br>⅄ LP.06 Six Leaves Visible<br>⅄ LP.12 Twelve Leaves Visible<br>⅄ LP.04 Four Leaves Visible<br>⅄ F Mature Embryo Stage<br>⅄ D Bilateral Stage<br>⅄ E Expanded Cotyledon Stage<br>⅄ LP.02 Two Leaves Visible<br>⅄ LP.10 Ten Leaves Visible<br>⅄ C Globular Stage<br>⅄ Leaf Development Stages<br>⅄ LP.08 Eight Leaves Visible
Contig 09781<br>nsSNP Position 386	⅄ Plant Structure Development Stage
