# Supplementary figures and images for: Predicting the Functional, Molecular, and Phenotypic Consequences of Amino Acid Substitutions using Hidden Markov Models
Source: Hum Mutat. 2012 Oct 3;34(1):57–65. doi: 10.1002/humu.22225 (PMC3558800; doi:10.1002/humu.22225)

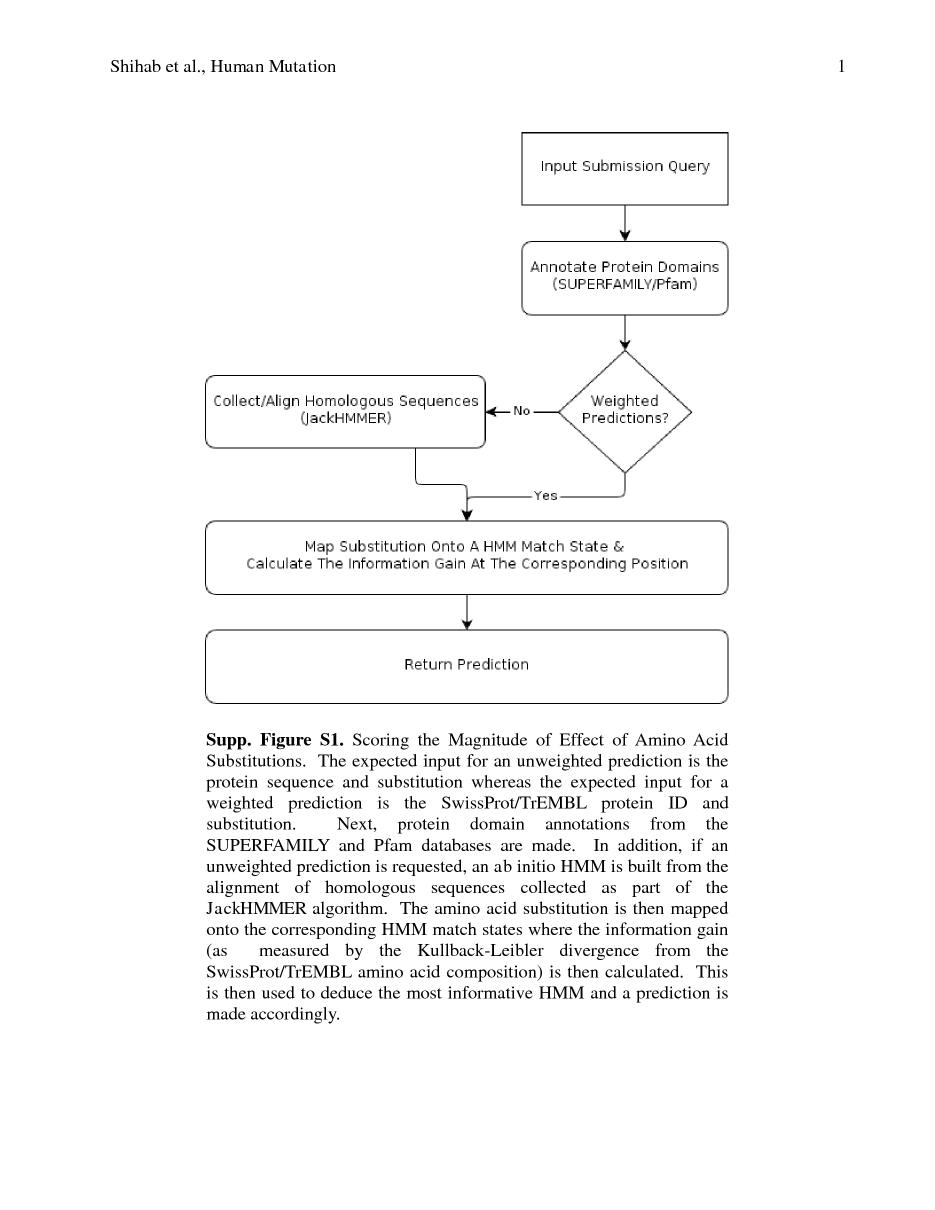

Supplement: Supplementary file 2 [file humu0034-0057-SD2.png]
